# Homeostatic medicine: a strategy for exploring health and disease

**DOI:** 10.1007/s44194-022-00016-9

**Published:** 2022-09-26

**Authors:** Songlin Wang, Lizheng Qin

**Affiliations:** 1 Beijing Laboratory of Oral Health, Capital Medical University, Beijing, 100069 China; 2 Salivary Gland Disease Center and Molecular Laboratory for Gene Therapy & Tooth Regeneration, Beijing Key Laboratory of Tooth Regeneration and Function Reconstruction, School of Stomatology, Capital Medical University, Beijing, 100050 China; 3 Department of Biochemistry and Molecular Biology, School of Basic Medical Sciences, Capital Medical University, Beijing, 100069 China; 4 Department of Oral and Maxillofacial & Head and Neck Oncology, School of Stomatology, Capital Medical University, Beijing, 100050 China

**Keywords:** Homeostasis, Homeostatic medicine, Nitric oxide (NO), Nitrate, Sialin

## Abstract

Homeostasis is a process of dynamic balance regulated by organisms, through which they maintain an internal stability and adapt to the external environment for survival. In this paper, we propose the concept of utilizing homeostatic medicine (HM) as a strategy to explore health and disease. HM is a science that studies the maintenance of the body’s homeostasis. It is also a discipline that investigates the role of homeostasis in building health, studies the change of homeostasis in disease progression, and explores ways to restore homeostasis for the prevention, diagnosis and treatment of disease at all levels of biological organization. A new dimension in the medical system with a promising future HM focuses on how homeostasis functions in the regulation of health and disease and provides strategic directions in disease prevention and control. Nitric oxide (NO) plays an important role in the control of homeostasis in multiple systems. Nitrate is an important substance that regulates NO homeostasis through the nitrate-nitrite-NO pathway. Sialin interacts with nitrate and participates in the regulation of NO production and cell biological functions for body homeostasis. The interactions between nitrate and NO or sialin is an important mechanism by which homeostasis is regulated.

## Introduction

Medicine is a systematic study of human life. The definition of life varies greatly, depending on one’s perspective, however, life is generally defined as an open system (Abel 2011) characterized by energy metabolism, stimulus-response compatibility, and self-reproduction. Basically, all living things are made up of matter, thus, any movement of living things should obey the general laws of physics as described in the second law of thermodynamics, that is, all systems have a spontaneous tendency towards disorder and a high-entropy state. For the system to maintain order and a low entropy state, the living body needs to constantly ingest substances with a high entropy state in the environment and convert them into a low-entropy state by consuming energy and excreting metabolic wastes (Chirumbolo and Vella 2021). Therefore, the activities of the living body involve a series of physical and chemical reactions, and these reactions must take place under the corresponding reaction conditions. To ensure the orderliness and efficiency of these reactions, an organism should maintain a relatively stable internal state, which is referred to as homeostasis. Organisms are in a constantly changing environment, but regardless of how the environment changes, they must maintain their internal stability for normal physiological functions to occur; such a process is called homeostasis regulation (Billman 2020).

Simply put, homeostasis is a self-regulating process through which organisms remain stable and constantly adapt to the changing external environment, thus leading to better survival. Health and disease are opposites that are interrelated with each other, and homeostasis regulation is the key factor in their mutual transformation. There are two aspects to health. One is that each system of the body maintains a normal physiological equilibrium while the other is that organisms are able to adapt to environmental changes, external stimuli, and pathogenic factors. Any disruption to the regular equilibrium regulation leads to disease, followed by a series of metabolic, functional, and structural changes that manifest as abnormal symptoms, signs, and behaviors (Lopez-Otin and Kroemer 2021). In other words, homeostasis is an intersection of health and disease; thus, good maintenance of homeostasis is a prerequisite for health, whereas disruption of homeostasis will inevitably lead to disease.

**Fig. 1:**
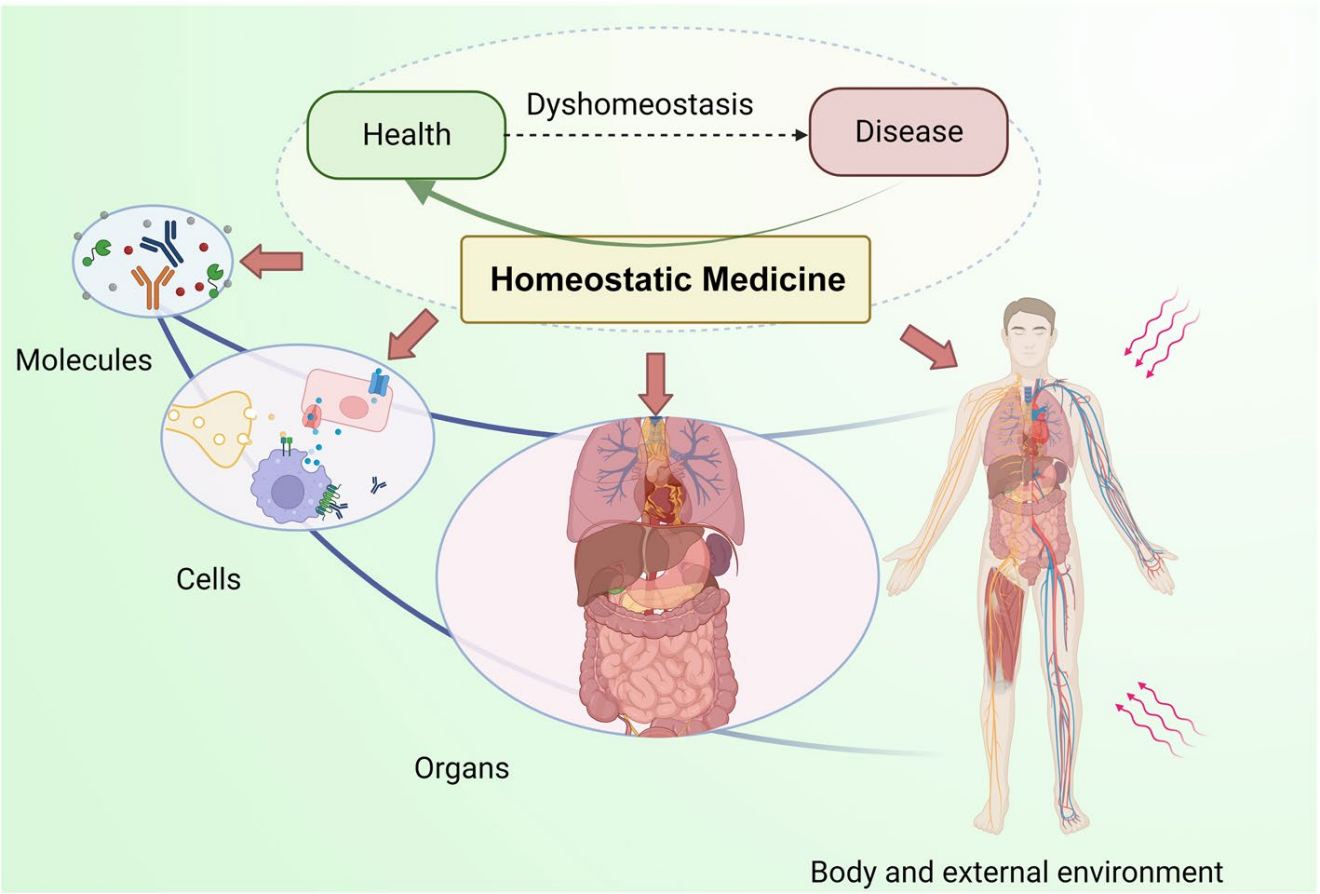
The conception of homeostatic medicine. Homeostatic medicine is a science of the homeostatic of molecules, cells, organs, and the whole body. Homeostasis is closely related to health and disease. The main idea of homeostatic medicine is to maintain human health and to prevent or treat diseases by modulating the homeostasis balance. Created with BioRender.com

Therefore, we propose the concept of homeostatic medicine, as a science of the homeostasis of molecules, cells, organs, and the whole body. Homeostatic medicine is a comprehensive discipline with the basic idea of maintaining human health and preventing and diagnosing diseases by maintaining the equilibrium of the internal environment required by the human system for survival **(**Fig. 1**)**. Homeostatic medicine focuses on the strategy of homeostasis regulation at the molecular, cellular, organ, systems, and external environmental levels. The ultimate goal of homeostatic medicine is to study the role of homeostasis in health and disease and to regulate homeostasis for the purpose of maintaining health and treating diseases. Different from traditional “symptomatic treatment,” homeostatic medicine focuses more on the destruction of homeostasis as the cause of the disease, which is “etiological treatment”. The idea of homeostatic medicine calls for further research on the relationship between health and disease and provides new ideas and strategies for health maintenance and disease prevention and treatment.

Homeostatology is a subject that focuses on the concept of homeostatic medicine but is not limited to biology or medicine. The research subjects of homeostatology are physics, psychology, and even sociology, which is a new scientific research idea and methodology for predicting and controlling various systems by studying self-stability phenomena. Homeostatology generalizes various self-stable phenomena in the energy transfer, information exchange and matter exchange and proposes the basic structure of system auto-stability. Briefly, antagonistic information exchange exists among the elements in the system, and the loop formed by this information exchange constitutes the fundamentals of equilibrium. Through homeostatology, people can better understand the principles of the world, and rational use of homeostatological methods is conducive to the research and progress of various disciplines.

## The history of the concept of homeostasis

The concept of homeostasis has experienced a dialectic period. The original theory of body equilibrium can be traced back to 460 BC when Hippocrates first proposed the Four Humors theory to describe the balanced state of the human body. He proposed that the human body consisted of four major fluids or humors, that is, blood, phlegm, yellow bile, and black bile. These four major humors must be maintained in equilibrium in order to promote good health (Santacroce et al. 2021). Compared to modern medicine, the Four Humors theory has many limitations, however, this theory remained for nearly two thousand years, influencing both Western and Eastern medicine. Hippocrates further suggested that the body can heal itself, and it is the doctor’s responsibility to clear obstacles to return the patient’s body to its natural state (Billman 2020). This view has been maintained until today, and for this theory, Hippocrates is regarded as “the father of Western medicine”.

With the constant exploration of body functions, the mechanism of homeostatic regulation has been further explained. French physiologist Claude Bernard (1813–1878) proposed the theory of “homeostasis of internal environment” (Adolph 1961). The theory holds that living systems have internal stability, which can buffer and protect the body from the constantly changing external environment. He stated that the human body was a collection of body fluids and cells in the internal environment and he believed that the stability and independence of the internal environment are necessary prerequisites for the survival of the body. However, Bernard’s viewpoint is flawed because he believed that the internal environment of the body was unchangeable and independent of the external environment, which was later proved to be inaccurate (Gross 2009).

Based on Claude Bernard’s theory of “homeostasis of internal environment,” Walter B. Cannon (1871–1945), an American medical scientist, improved this theory by introducing the concept of dynamic balance. Dynamic balance is a self-regulation process indicating that organisms can maintain stability while adapting to the changing environment (Cooper 2008). In Cannon’s book, *The Wisdom of the Body*, he explained that homeostasis requires two components at the same time: internal stability within a certain range and the ability to remain stable by regulating variables (Billman 2020). Canon’s theory of homeostasis has been recognized in modern medicine and has elaborated a good theoretical foundation for the study of homeostasis.

Cybernetics was first proposed by Norbert Wiener (1894–1964) in 1948. He defined cybernetics as a science that focuses on general laws of control and communication in machines, life and society. Its core idea is to study how dynamic systems maintain equilibrium under the changing environment (Wiener 1948). It has been widely applied to a variety of complex control systems with multiple factors, such as the economic regulation system, the resource allocation system, the ecological and environmental system, and the computer logic system. The feedback control theory in cybernetics refers to a regulation process in which the difference between the result and the standard is identified through information feedback and corrective measures are taken to stabilize the system at the target state, which corresponds to the negative feedback regulation mechanism in homeostasis regulation. Moreover, the adaptive theory refers to the automatic adjustment of its own structure or behavior parameters according to the changes of external conditions before the environmental conditions have affected the control object, so as to maintain the original function of the system, which corresponds to the feed-forward adjustment mechanism in homeostasis regulation (Benjamin et al. 2020).

Similar to the concept of homeostasis in Western medicine, traditional Chinese medicine pursues the theory of Yin and Yang. Yin and Yang in Equilibrium and The Pursuit of Balance were first proposed in *Huangdi’s Class*ic on Medicine (Huang Di Nei Jing). Yin and Yang in Equilibrium implies opposing and restricting forces, which eventually harmonize to form a relative dynamic balance. The Pursuit of Balance emphasized the importance of maintaining a balance. Therefore, it is more important to restore balance than to remove the causes of a disease. Furthermore, excessive treatment should be avoided so as not to break the balance between Yin and Yang (Maiese 2006). Clearly, though there is great difference in Eastern and Western medicine, the understanding of the role of homeostasis in health and disease is quite close.

At the beginning of the life circle, the regulation of homeostasis plays an important role in organisms. It would not be exaggerating to say that homeostasis regulation distinguishes living things from non-living things. It provides a basis for life to maintain a state of low entropy, and is an important mechanism of evolution in organisms. The prevailing view is that life began in the ocean. The ancient ocean was a potassium-rich environment, which can be viewed as a precursor to potassium-rich intracellular fluids. As the environment evolved, the ocean changed into a sodium-rich environment, which is viewed as a precursor to extracellular fluid. The existence of cell membranes and sodium/potassium pumps provides the structural basis for the homeostasis regulation of potassium ions and the membrane potential in cells and makes it possible to conduct electrical signals in nerves and muscles (Sieck 2017). As the structure of living organisms became more complex, single-celled organisms began to metabolize and cooperate to better maintain the low entropy state of the system. In prokaryotes, biofilm systems and a sense of community emerged, while in eukaryotes, primitive intercellular signaling systems emerged, and this primitive homeostasis regulatory mechanism underlies morphogenesis, dynamic homeostasis, regeneration, and reproduction (Torday 2013). Thus, homeostasis regulation is involved in each key node of biological evolution and is an important factor in promoting the evolution of organisms.

## Homeostasis regulation

The most important mechanism in homeostasis regulation is the feedback system, which has four main components: (1) the variable to be controlled, (2) a sensor that monitors the variable, and (3) a comparator or central processing unit where the information provided by the sensor is fed back into the system. The information is compared with the specified value, and (4) effectors are used to regulate the desired control variables (Billman 2020). These parts form a closed loop that feeds the signal back. In a negative feedback process, the activity of the effector is opposite to the changes in the variables to buffer them. In a positive feedback process, the activity of the effector is identical to its changes to achieve rapid changes in the state of the body by amplifying a control signal (Goldstein and Kopin 2017). Nevertheless, it is noteworthy that the four components only represent the vitals of the feedback system; the feedback system in biology is much more complicated, involving the superposition and nesting of multiple feedback pathways.

Taking blood pressure as an example, most of the time, our blood pressure is maintained within a relatively stable range through feedback regulation. The receptors for blood pressure in the body are baroreceptors on the aortic arch and carotid sinuses which respond to changes in arterial pressure. The solitary tract in the medulla oblongata of the brain processes signals from pressure receptors and acts on the effectors located in the blood vessels and heart by regulating the neural activity of the sympathetic and parasympathetic nerves (Dworkin and Dworkin 1995). When the blood pressure is elevated, the baroreceptors are activated, and the regulation of the solitary tract nucleus decreases sympathetic activity and increases the vascular diameter. In addition, increased parasympathetic activity lowers the heart rate and stroke output, thus lowering the blood pressure. The opposite occurs when the blood pressure is below a set point. Through this negative feedback regulation, fluctuations in blood pressure can be effectively buffered so that the body’s blood pressure can remain relatively stable all day, despite changes in the environment or behavior (Humphrey and Schwartz 2021).

Feed forward regulation, another important mechanism of homeostasis regulation, refers to the assessment and adjustment of impending changes before they actually occur (Carpenter 2004). Several levels of regulation are involved in homeostasis. The first level is the effector responsible for receiving higher-level regulatory signals and variables. Feedback regulation, also known as autonomic regulation, is the second level of this system, which processes signals detected by receptors and initiates adjustments to the first level. The third level is located in the central nervous system, which processes information transmitted from the second level. It can integrate the changing information of the environment to coordinate the physiological behavior of various feedback systems (Goodman 1980). Under certain conditions, this regulation can be unconscious or regulated by subjective consciousness. Taking blood pressure regulation as an example, when faced with danger or challenge, the cardiac output rate and blood pressure can be raised through the central nervous system to respond to impending environmental changes, the process of which is not regulated by consciousness. However, when faced with cold weather, clothes are actively added to maintain body temperature. In this case, the regulation process is completed through the intervention of consciousness and experience (Billman 2020).

Homeostasis is a key regulatory mechanism in health and disease. Normal homeostasis is fundamental for maintaining health and ensuring various physiological functions. In contrast, disease progression is typically accompanied by an imbalance in homeostasis. These changes have adverse effects on the body and eventually cause functional disorders and organic lesions. As mentioned above, each physiological indicator is maintained within a prescribed range through homeostasis regulation. However, this range is not fixed, and the body can adjust the default physiological indicators to adapt to different needs under certain circumstances (Kotas and Medzhitov 2015). For example, the default setting point for body temperature is approximately 37 °C. In conditions of inflammation or infection, the setting point of the temperature can increase to 40 °C to increase the basal metabolic rate and defend against infection, a process commonly known as “fever” (Morrison 2016). When the external stimuli are removed, the setting point of the body temperature gradually returns to normal. In this case, fever helps the body to cope with extreme challenges. However, prolonged high fever can cause structural and functional destruction of tissues and organs and, in severe cases, multiple organ failure and even death. In short, when the adjustment of the setting point deviates from the acceptable range, it causes irreversible effects on the homeostasis of the body, leading to a pathological state.

Homeostatic medicine is the science of the homeostasis of molecules, cells, organs, and the whole body. The basic idea is to maintain human health, to prevent or treat diseases by maintaining homeostasis. Homeostatic medicine systematically studies the mechanism of homeostasis regulation and summarizes a series of strategies to guide clinical treatment. Homeostatic medicine integrates the concept of homeostasis in both Eastern and Western medicine and summarizes the role of homeostasis regulation in health and disease. The destruction of homeostasis is the essence of disease. Therefore, homeostatic medicine mainly focuses on restoring homeostasis to eliminate the cause of disease. The goal of homeostatic medicine is to study changes in homeostasis in disease and to incorporate existing medical methods to treat or alleviate the disease **(**Fig. 1**)**. Homeostatic medicine comprises three key steps. The first is to understand the mechanism of homeostasis regulation and its role in maintaining health. The next is to analyze the causes and intervention factors of homeostasis imbalance in the process of disease. Finally, it consolidates the information gained in the first two steps and restores the homeostasis of the body through reasonable interventions. Given the crucial role of homeostasis in health and disease, homeostatic medicine has a wide range of applications in coping with various diseases.

## Fifty percent concept in homeostasis

The reserve function of organisms is an important mechanism for homeostasis regulation, which is reflected in all levels of the body. Approximately 50% of the physiological potential of the body is not utilized, which is known as the reserve function of the body. Only in response to abnormal conditions or external challenges can the physiological potential of surplus reserves be mobilized, which makes it possible for the organism to deal with overload work, thus facilitating the rapid recovery of homeostasis (Atamna et al. 2018). The reserve function of an organism is closely related to its health and disease states. For example, aging causes a decline in the reserve capacity of the body’s tissues and organs, including the functional decline of the immune, muscular, and nervous systems. This reduction in reserve function leads to the decreased ability to respond to external stimuli, increased susceptibility to infection, and a longer recovery time from diseases (Iliodromiti et al. 2016).

The metabolic efficiency of an organism depends on the number and activity of enzymes, and excessive enzymes are the basis of the metabolic reserve. For example, with an excessive quantity of glycolytic enzymes, the body can respond to the energy demand of overloaded physiological activities and increase these to the maximum capacity when necessary to maintain homeostasis (Eanes et al. 2006). In nerve cells, neuronal activity depends on the maintenance of the membrane potential by sodium/potassium ATP pumps. The sodium/potassium ATP pump shows a large reserve capacity to ensure neuronal homeostasis under highly intensive neural activity (Howarth et al. 2012). In addition, the amount of mitochondrial DNA (mtDNA) in a cell is much higher than the number of mitochondria. An average of 2000–10,000 copies of mtDNA is estimated to be contained in a nucleated cell with 1000 mitochondria. This redundant mtDNA has a reserve capacity that can reduce the impact of mutations on energy metabolism through the excess mtDNA reserves (Miller et al. 2003). The regulation of reserve function in homeostasis can also be reflected at the cellular level. In a healthy state, most cells of the human body are in a static state of division. In response to adverse stimuli, these static cells can quickly enter the cell cycle to repair damaged tissues and functions. For example, in the process of liver regeneration after toxic injury or hepatectomy, epithelial and parenchymal cells can respond quickly, rapidly divide, and differentiate to restore the quality and function of the liver (Campana et al. 2021). At the organ level, in the case of the kidney, the concept of the renal functional reserve has been proposed for decades, which refers to the difference between the glomerular filtration rate at rest and at maximum capacity. In general, utilization of the kidney accounts for only approximately 50% of the total potential. The renal filtration rate is considerably increased in pregnancy, isolated kidney, or hypertensive diabetic nephropathy, and the presence of the renal reserve allows the serum creatinine and glomerular filtration rate to remain normal in the presence of renal damage until more than 50% of renal units are lost (Palsson and Waikar 2018).

The effect of the reserve function on homeostasis is one of the focuses of homeostatic medicine. Making full use of the 50% reserve potential to maintain a steady state is an important scientific issue. Sometimes, not only is it difficult to completely cure a disease or restore the body to a healthy level, but also excessive treatment can cause serious side effects. Benefiting from the reserve function, healthy homeostasis can theoretically be maintained by restoring 50% of the function of the body or by maintaining half of the organ’s health. For example, in kidney transplant donors, a single kidney is sufficient to meet normal functional demands through compensation (Steiger 2011). Therefore, it may be more reasonable to aim for the health of one kidney than the more difficult goal of two healthy kidneys. The previous concept states that health occurs only if all organs are intact. In contrast, homeostatic medicine adheres to the 50% treatment concept, the goal of which is to restore at least 50% of physiological function and to maintain homeostasis by making the most use of reserve function.

## Nitrogen oxygen and Sialin-two important regulators in homeostasis

**Fig. 2:**
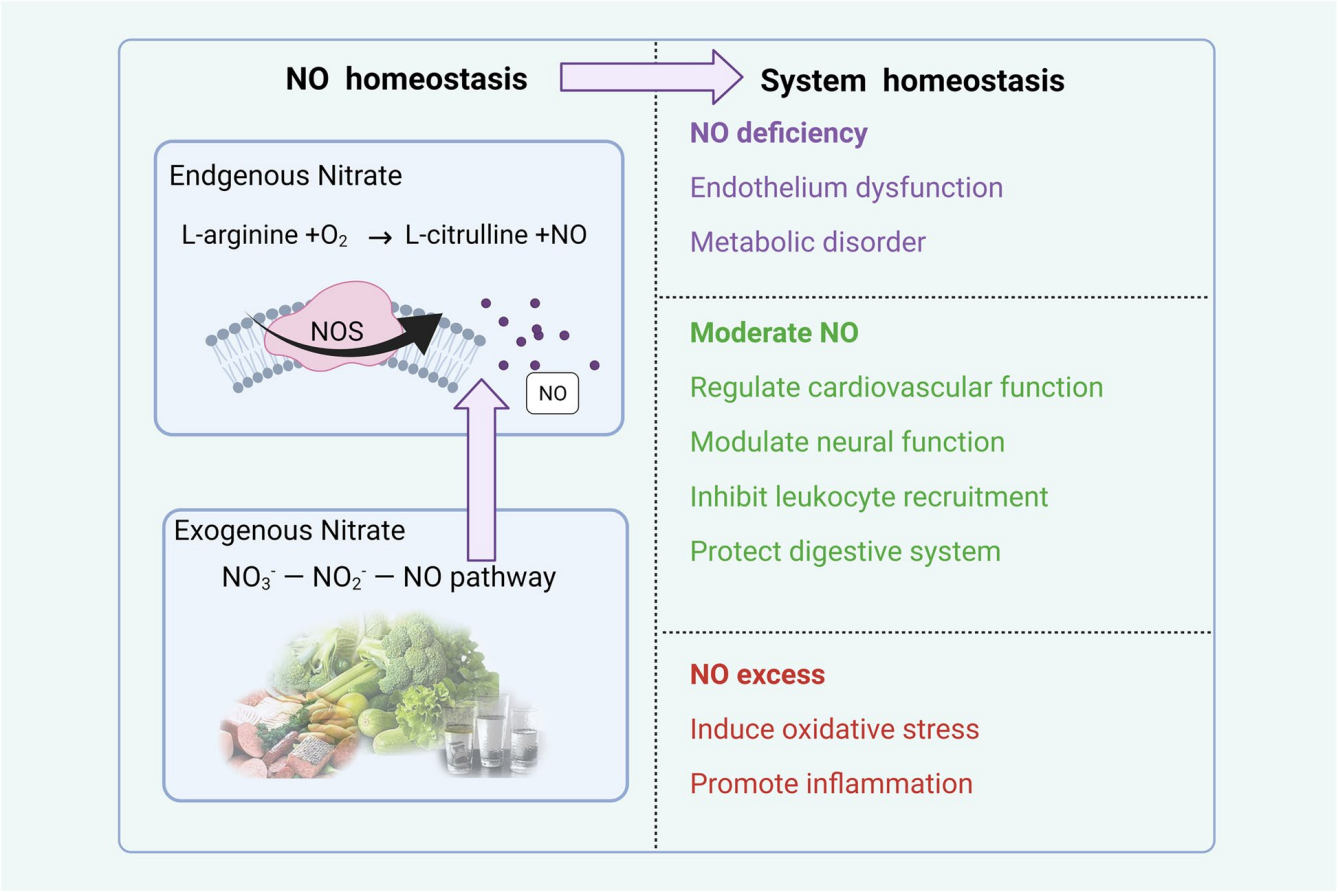
The relationship between nitrate, NO homeostasis. and system homeostasis. NO is an important molecule to maintain body homeostasis. Through the synthesis of endogenous NO by nitric oxide and enzymes and the intake of exogenous nitrate, the level of NO in the body can be maintained in a relatively stable range. The homeostasis of NO is of great significance to the homeostasis of the body. Insufficient NO will cause endothelial dysfunction and metabolic disorder, while excessive NO will induce oxidative stress and promote inflammation. Created with BioRender.com

Nitric oxide (NO) is perceived as a harmful atmospheric pollutant. However, Furchgott et al. proposed that NO is a signaling molecule in the cardiovascular system, which won the Nobel Prize in 1998. Since then, it has been noted that NO is an important signaling molecule in mammals and plays a variety of important physiological functions in various systems of the body, including the nervous, digestive, urinary, reproductive, immune, and cardiovascular (Moncada et al. 1991). As an important molecule in homeostasis regulation, NO plays a key role in maintaining homeostasis in the body. For example, as signaling molecules, NO can regulate the dynamic balance of the endocrine system by regulating the neuroendocrine and autonomic nervous systems. In the case of dehydration and bleeding stress, NO plays a protective role and restores the autonomic neuro-humoral balance (Krukoff 1999). However, in some cases, NO plays a dual role. For example, NO can inhibit tissue inflammation by inhibiting leukocyte recruitment (Kubes et al. 1991). However, excessive NO production will cause oxidative stress in cells and further promote inflammation (Krol and Kepinska 2020). Therefore, maintenance of NO homeostasis in vivo contributes to the regulation of systemic homeostasis. NO is synthesized internally by nitric oxide synthase (NOS). Moreover, when endogenous NO synthesis is impaired, such as in endothelial dysfunction, exogenous nitrate intake can effectively maintain NO homeostasis (Kapil et al. 2020a) **(**Fig. 2**)**.

**Fig. 3:**
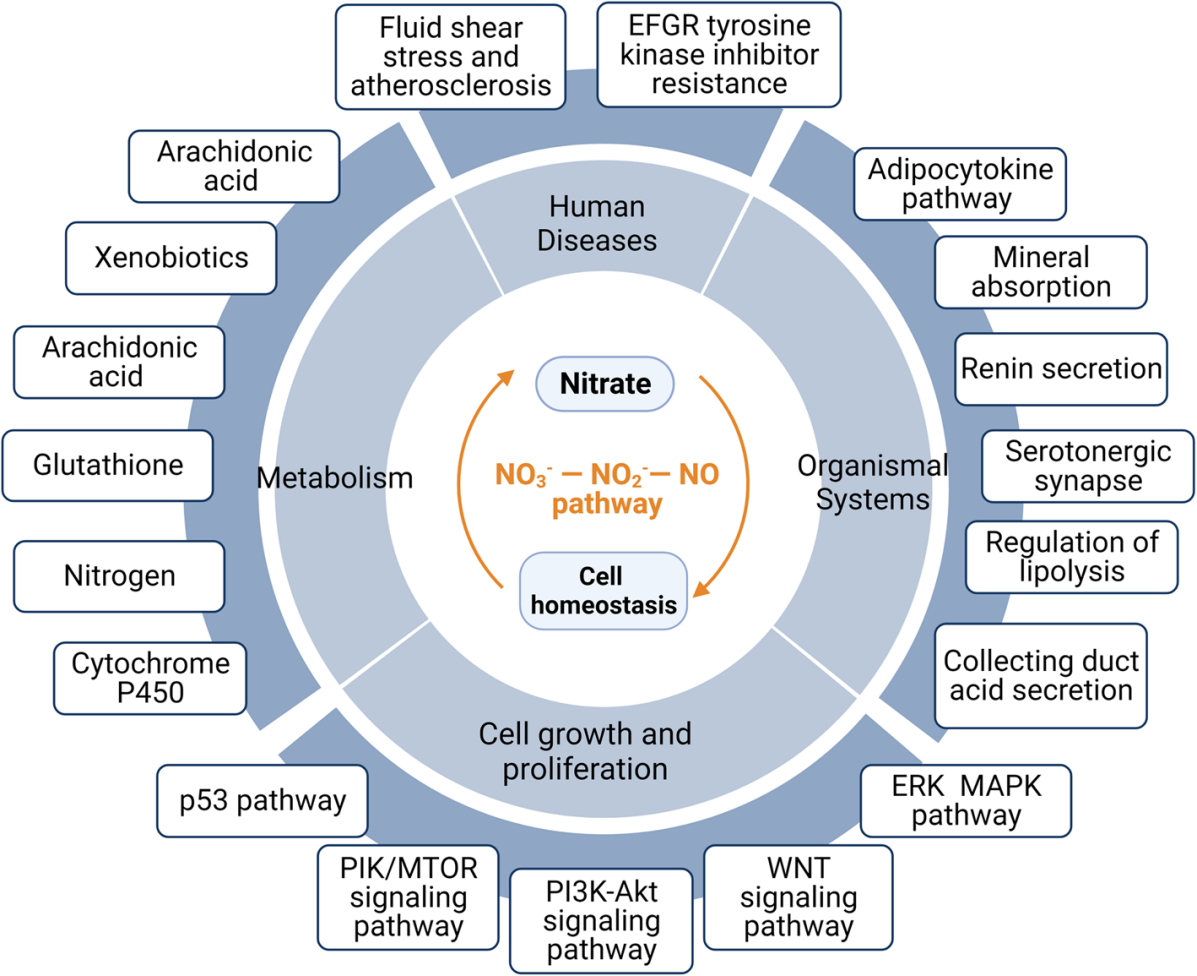
Signaling pathways and functions of nitrate on the regulation of homeostasis. Through the nitrate-nitrite-NO pathway, nitrate plays an important role in regulating homeostasis of cells from multiple perspectives, including regulating metabolism, promoting cell regeneration, and inhibiting disease progression

Nitrate is widely distributed in nature, particularly in green vegetables. After a nitrate-rich meal, nitrate is rapidly absorbed into the blood in the upper digestive tract. Approximately 25% of the nitrate is absorbed by the salivary glands and secreted into the oral cavity by the saliva. This process is called intestinal salivary circulation, which is conducive to maintaining nitrate levels in vivo. Nitrate in the saliva is reduced to nitrite by nitrate-reducing bacteria inhabiting the oral cavity. These nitrites are then swallowed and reabsorbed into the blood, where they can be reduced to NO by various enzymes in the blood and tissues. This process is known as the nitrate-nitrite-NO pathway. Through the nitrate-nitrite-NO pathway, nitrate performs a variety of NO-like physiological functions (Ma et al. 2018), including protecting the digestive system by increasing blood flow and regulating intestinal flora (Hu et al. 2020a; Wang et al. 2020a), alleviating obesity by adjusting fat metabolism (Ma et al. 2020a) and assisting tumor therapy by increasing sensitivity to cisplatin chemotherapy (Feng et al. 2021a). Moreover, nitrate can significantly upregulate the expression of a variety of cellular signaling pathways, including the MAPK signaling pathway (Feng et al. 2021b), the PI3K-Akt signaling pathway, the mTOR and WNT signaling pathway (Jiang et al. 2014), glutathione metabolism, and the cell cycle (Jia et al. 2011), which play important roles in cell regeneration, cell metabolism, and disease progression **(**Fig. 3**)**.

**Fig. 4:**
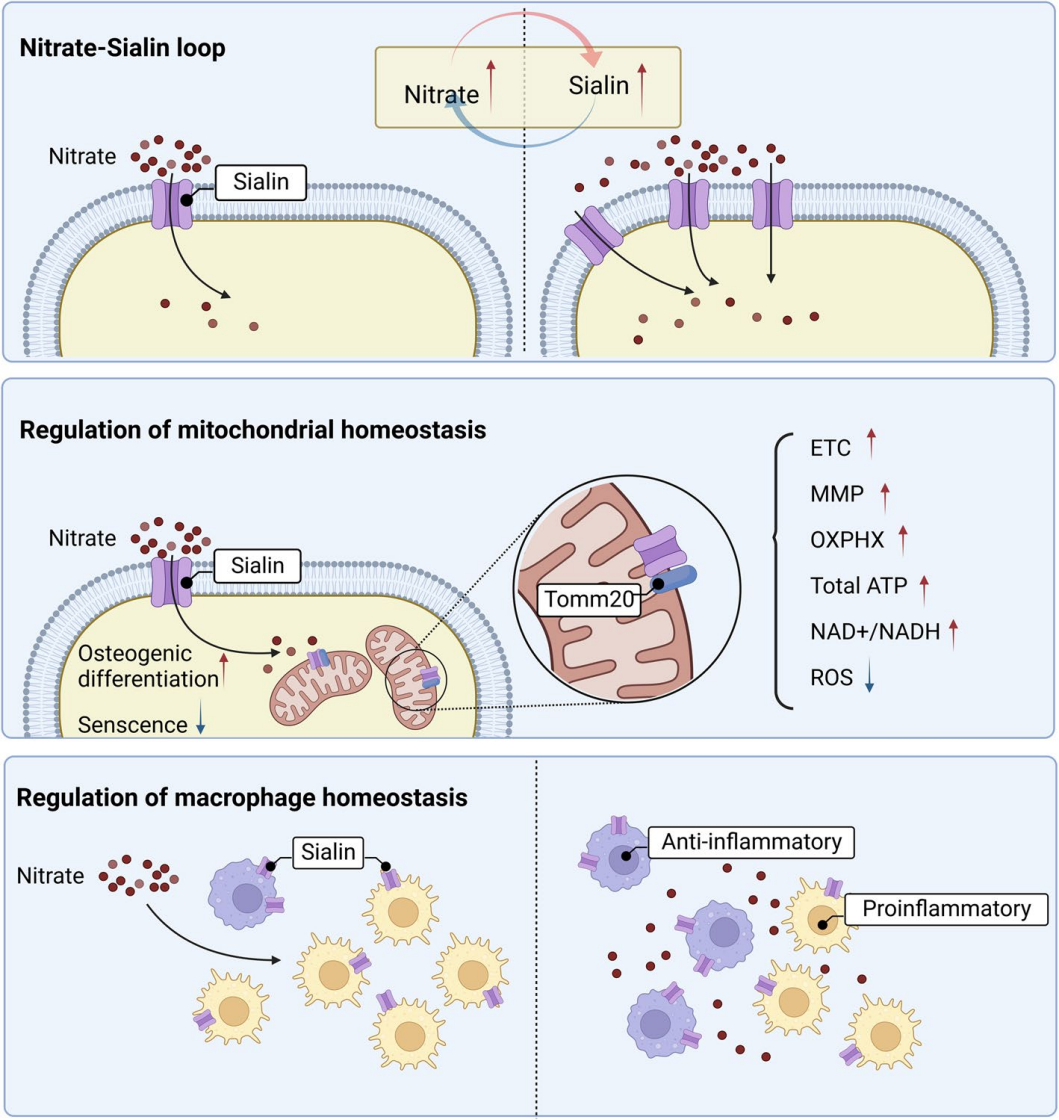
The mechanism of sialin on the regulation of homeostasis. Sialin is a membrane transporter of nitrate and plays an important role in homeostasis regulation. There is a positive feedback loop between nitrate and sialin, which is beneficial for nitrate to enter cells. Sialin can also regulate the function of mitochondria to improve the antioxidant stress ability of mesenchymal stem cells. Moreover, sialin is involved in the regulation of inflammatory phenotypes of macrophages to prevent non-alcoholic fatty liver disease. Created with BioRender.com

Sialin is a nitrate transporter in mammalian cell membranes that plays a key role in the beneficial effects of nitrate and homeostasis maintenance of NO (Qin et al. 2012). There is an interaction between nitrate and sialin, which is usually accompanied by elevated sialin expression in important organs. The high expression of sialin promotes a range of cellular biological functions. Nitrates improve mitochondrial function and reduce aging of mesenchymal stem cells by upregulating sialin expression. Oral administration of inorganic compounds can directly regulate the M1/M2 ratio of macrophages, thus preventing non-alcoholic fatty liver disease. In addition, sialin is highly expressed in human salivary glands, and salivary gland function is an important factor in the physiological function of nitrate (Qu et al. 2016). Interestingly, nitrate can regulate salivary gland function. In rats with xerostomia caused by ovariectomy, inorganic nitrate upregulated the expression of aquaporin-5 in the salivary glands, thus increasing salivary secretion and effectively reducing the fibrosis area and acinous atrophy of salivary gland tissues (Xu et al. 2018). In a salivary gland radiation injury model of miniature pigs, nitrate increased the expression of sialin in acinar cells, which further promoted the entry of nitrate into cells. This nitrate-sialin loop activates the EGFR-Akt-MAPK signaling pathway, thereby promoting the proliferation of acinar and ductal cells and reducing apoptosis (Feng et al. 2021b). The nitrate-sialin loop preserves radiation-lost salivary gland cells by regulating autophagy within salivary gland cells. It follows that the mutual effects of sialin and nitrate are beneficial for maintaining NO homeostasis and further improving systemic homeostasis **(**Fig. 4**)**. Therefore, the nitrate-sialin loop and the regulatory effect of nitrate on NO homeostasis may be a promising research direction, and the mechanism by which nitrate regulates systemic homeostasis deserves further study.

## Homeostasis and disease

### Homeostasis in tumors

The occurrence and development of tumors are closely related to dyshomeostasis. Proto-oncogenes are a group of genes that play important roles in cell proliferation and differentiation. Normally, proto-oncogenes are strictly regulated by another group of genes called tumor suppressor genes, and the dynamic balance between them is conducive to maintaining the number of cells and normal physiological function. However, the balance between proto-oncogenes and tumor suppressor genes can be disrupted by gene mutations under the action of stimulating factors, and the overexpression of proto-oncogenes may lead to abnormal cell proliferation and tumor formation (Pitot 1993). At present, the mainstream treatments for cancer are surgery, radiotherapy, chemotherapy, and immunotherapy, which aim to reduce tumor volume, block its invasiveness, and induce tumor cell death. Homeostasis regulation is an important factor in tumor occurrence and development. Therefore, homeostatic medicine aims to combine the concept of homeostasis regulation with tumor treatment. It can improve the resistance of normal tissues and organs to tumors by restoring their original homeostasis or inhibiting the growth of tumors by destroying the homeostasis of tumor tissue to improve the therapeutic effect on tumors and obtain twice the result with half the effort. In addition, it is controversial whether all tumors in the body must be eliminated for the sole purpose of treatment. Considering the negative effects of total tumor removal, one of the therapeutic goals should be to control tumor growth and maintain homeostasis in the body.

Inorganic nitrate can improve the sensitivity of oral squamous cell carcinoma to chemotherapy by decreasing the expression of REDD1 (regulated in development and DNA damage responses 1), which may be associated with improved NO homeostasis in hypoxic tumor tissues (Feng et al. 2021a). Inorganic nitrates also showed considerable protective effects against the side effects of tumor radiotherapy. Inorganic nitrate effectively relieved the decline of salivary gland function after radiation injury in rats (Feng et al. 2021b; Li et al. 2021a), and it relieved colitis in rats after systemic radiation by regulating the homeostasis of intestinal flora (Wang et al. 2020b). These results suggest that inorganic nitrates may improve the postoperative quality of life of patients with tumors after radiotherapy. The anti-radiation effects of nitrate are not limited to this. Even at low radiation intensities, such as after cone-beam computed tomography (CBCT) examination, nitrate shows an inhibitory effect on radiation-induced oxidative stress (Yang et al. 2022).

### Homeostasis in cardiovascular disease

Homeostasis plays a key role in the health and disease of the cardiovascular system. Mechanobiological stability can be maintained in healthy blood vessels through multiple levels of negative feedback. However, the progression of cardiovascular disease is usually associated with overregulation of the mechanical biological balance or unstable positive feedback (Humphrey and Schwartz 2021). Generally, the cardiovascular system can adapt to changes in the internal and external environments of the body through functional and structural regulation. For example, a continuous increase in cardiac output causes dilation of the central artery, which reduces resistance to blood flow and thus reduces the heart’s workload. These regulations are beneficial for maintaining homeostasis (Humphrey 2008). However, long-term constriction or dilation of blood vessels causes excessive dilation and thinning of blood vessels, even aneurysm or arterial rupture. To enhance vascular stress intensity, blood vessels begin to develop fibrosis. Eventually, excessive fibrosis of the blood vessels leads to vascular sclerosis and hemadostenosis. These changes cause atherosclerosis and hypertension, which further increase stress on the cardiovascular system (Schwartz et al. 2018). This abnormal positive feedback is one of the pathogenic mechanisms underlying cardiovascular disease.

At present, the treatment of cardiovascular disease mainly focuses on alleviating the symptoms of patients. These include reducing the blood volume by using diuretics, limiting vasoconstriction by inhibiting the renin-angiotensin pathway and calcium channels, or lowering cholesterol synthesis to alleviate atherosclerosis by using statins (Takebe et al. 2018). Although these treatments have been widely used in clinical practice and have achieved a certain efficacy, many drugs may cause unexpected side effects that are not conducive to their long-term application.

Ideally, homeostatic treatment should comprehensively consider the mechanisms of vascular biomechanics, cell signal transduction, immunobiology, and other aspects of homeostasis regulation to avoid the disruption of homeostasis. By means of identifying and blocking therapeutic targets of disease processes, homeostatic medicine improves the state of a disease by restoring the dynamic balance of the cardiovascular system. Inorganic nitrate can maintain nitric oxide homeostasis through the nitrate-nitrite-nitric oxide pathway and has shown beneficial effects in a variety of cardiovascular diseases (Kapil et al. 2020b). A significant antihypertensive effect was still observed after 1 year of use of inorganic nitrate, proving that inorganic nitrate does not have the same drug resistance as organic nitrate (Munzel et al. 2005). Moreover, no adverse effects such as syncope or postural hypotension were observed during nitrate use. In addition, compared to healthy volunteers, more significant hypotensive effects of inorganic nitrate were observed in hypertensive patients with impaired endothelial function, suggesting that inorganic nitrate may contribute to ameliorating endothelial homeostasis disorders (Kapil et al. 2015).

### Homeostasis in metabolism diseases

Energy balance is mainly regulated by the central nervous system, including the neuroendocrine center located in the hypothalamus and the solitary nucleus located in the upper brainstem (Matafome and Seica 2017). Obesity is a metabolic disorder characterized by the excessive accumulation of body fat and is associated with an increased prevalence of various diseases. Metabolic homeostatic disorders are the most important causes of obesity (Lustig et al. 2022). Normally, the foraging behavior of the human body is dynamically balanced, and the desire to consume food increases to meet physiological needs when energy stores decrease. However, obese patients often exhibit a state of hedonism. To promote sensory pleasure, they consume food beyond energy balance, even when energy reserves are sufficient (Rossi and Stuber 2018). In addition, overeating alters the sensitivity of the dietary reward center to dopamine, further exacerbating the disruption of energy metabolism homeostasis and promoting the progression of obesity (Kessler et al. 2016).

Homeostatic medicine improves metabolic diseases by understanding the mechanism of homeostasis imbalance in metabolic diseases to eliminate the imbalance between energy uptake and metabolism. Inorganic nitrates ameliorate metabolic diseases, and their beneficial effects may be related to the promotion of the nitrate-nitrite-NO balance, thus maintaining NO homeostasis and regulating microbial homeostasis. In eNOS-deficient mice with impaired NO synthesis, inorganic nitrates decreased body fat and improved glucose homeostasis (Carlstrom et al. 2010). Liver senescence and metabolic dysfunction are also important causes of metabolic disease. Daily intake of nitrate can effectively restore the liver metabolic capacity of d-galactose-induced aging mice and naturally aging mice by preventing the aging of liver cells and the degeneration of glucose and lipid metabolism (Wang et al. 2018). Moreover, by activating the nitrate-nitrite-NO pathway, inorganic nitrates can promote the conversion of white adipose tissue to brown adipose tissue (Roberts et al. 2015). Inorganic nitrates can also reduce high-fat diet-induced obesity in mice and ameliorate glucose and lipid metabolism disorders by activating the NO pathway and regulating gut microbiota (Ma et al. 2020b).

### Homeostasis in immune and infectious diseases

The immune system, which can maintain homeostasis by immune surveillance, sensing metabolic changes, and controlling inflammation caused by external stimuli, is the most important line of defense of the body against harmful external stimuli and antigens (Weisberg et al. 2021). The body immunity maintains a dynamic balance under the interaction of various cellular and humoral immunities. Disorder in immune homeostasis is closely related to disease, and the development of a disease is the result of immune regulation failure (Kennedy 2010).

Take COVID-19 circulating worldwide as an example, which also disrupts the homeostasis of the immune system. COVID-19 is an infectious disease caused by SARS-COV-2 and characterized by acute respiratory failure. After infection, cells infected with SARS-COV-2 are recognized by macrophages and dendritic cells. Macrophages produce a large number of proinflammatory cytokines and chemokines, leading to the recruitment of inflammatory cells such as neutrophils to the site of the infection. The over release of pro-inflammatory cytokines may lead to cytokine storms, which can cause tissue damage, organ failure, and death. Severe symptoms are most common in elderly patients and are often accompanied by complications, while the symptoms in young people are often mild. This is because the homeostasis regulation of the immune system changes with the aging of the body. Compared to young patients, elderly patients have a lower response and clearance ability to viral infections, while the inflammatory response to infections is more active. Immune features are the main reason for the high severity and mortality of COVID-19 in elderly patients (Fulop et al. 2017). Homeostasis also plays an indispensable role in autoimmune diseases. Immune cells regulate and restrict each other to maintain the homeostasis of the immune system. For example, regulatory T cells can limit the activity of effector T cells, and reduced function of regulatory T cells can lead to autoimmune responses of effector T cells to tissues, such as rheumatoid arthritis (Kotschenreuther et al. 2022).

Dietary nitrate pretreatment can deliver NO to the vascular system and reduce the inflammatory response of leukocytes to acute chemokines through the nitrate-nitrite-NO pathway (Jadert et al. 2012). In mice susceptible to atherosclerosis, inorganic nitrate intake rescued NO homeostasis, thus inhibiting neutrophil activation and increasing IL-10 levels. Nitrate reduced the number of macrophages in atherosclerotic plaques and inhibited their inflammatory activity by reversing NO deficiency caused by endothelial dysfunction (Khambata et al. 2017). In liver oxidative stress injury induced by ischemia-reperfusion, inorganic nitrate intake increased NO levels in the plasma and liver and relieved liver oxidative stress by upregulating nuclear factor erythroid 2-related factor 2 (NRF2)-related molecules and increasing antioxidant enzyme activity (Li et al. 2021b). In addition, the homeostasis of microorganisms in the human body has important regulatory roles in infectious diseases as well as in inflammatory processes. Inorganic nitrates can regulate the homeostasis of intestinal bacteria, alleviate dextran DSS-induced enteritis, improve colon length, and maintain the body weight of mice (Hu et al. 2020).

In summary, homeostasis regulation plays an important role in the health maintenance and disease prevention in different systems. Any disruption of homeostasis causes body dysfunction and ultimately leads to disease. Therefore, restoring the body’s homeostasis is key to reversing disease states. Homeostatic medicine integrates the mechanisms of homeostasis regulation at the molecular, cellular, organ, and systemic levels. By understanding the regulatory mechanism of homeostasis in health and disease states, the imbalance in homeostasis can be reversed through reasonable intervention. In this way, homeostatic medicine contributes to the maintenance of health and the treatment of disease. Homeostatic medicine, which has a very broad development prospect, is a new medical system that is expected to provide a new strategy for medical research and disease treatment. Regulation of NO homeostasis is closely related to the homeostasis of multiple systems in vivo. Inorganic nitrate, which can provide a variety of beneficial physiological functions through the nitrate-nitrite-NO pathway, is an important substance that regulates NO homeostasis. As a key protein for nitrate to enter cells, sialin interacts with nitrate and participates in the regulation of NO production and body homeostasis. Sialin can independently mediate a range of cellular functions. Therefore, the interaction between NO, nitrate, and sialin is an important mechanism for homeostasis regulation, which is of great concern in the study of homeostatic medicine.
